# Supplementary figures and images for: Structure of G protein-coupled receptor GPR1 bound to full-length chemerin adipokine reveals a chemokine-like reverse binding mode
Source: PLoS Biol. 2024 Oct 28;22(10):e3002838. doi: 10.1371/journal.pbio.3002838 (PMC11515964; doi:10.1371/journal.pbio.3002838)

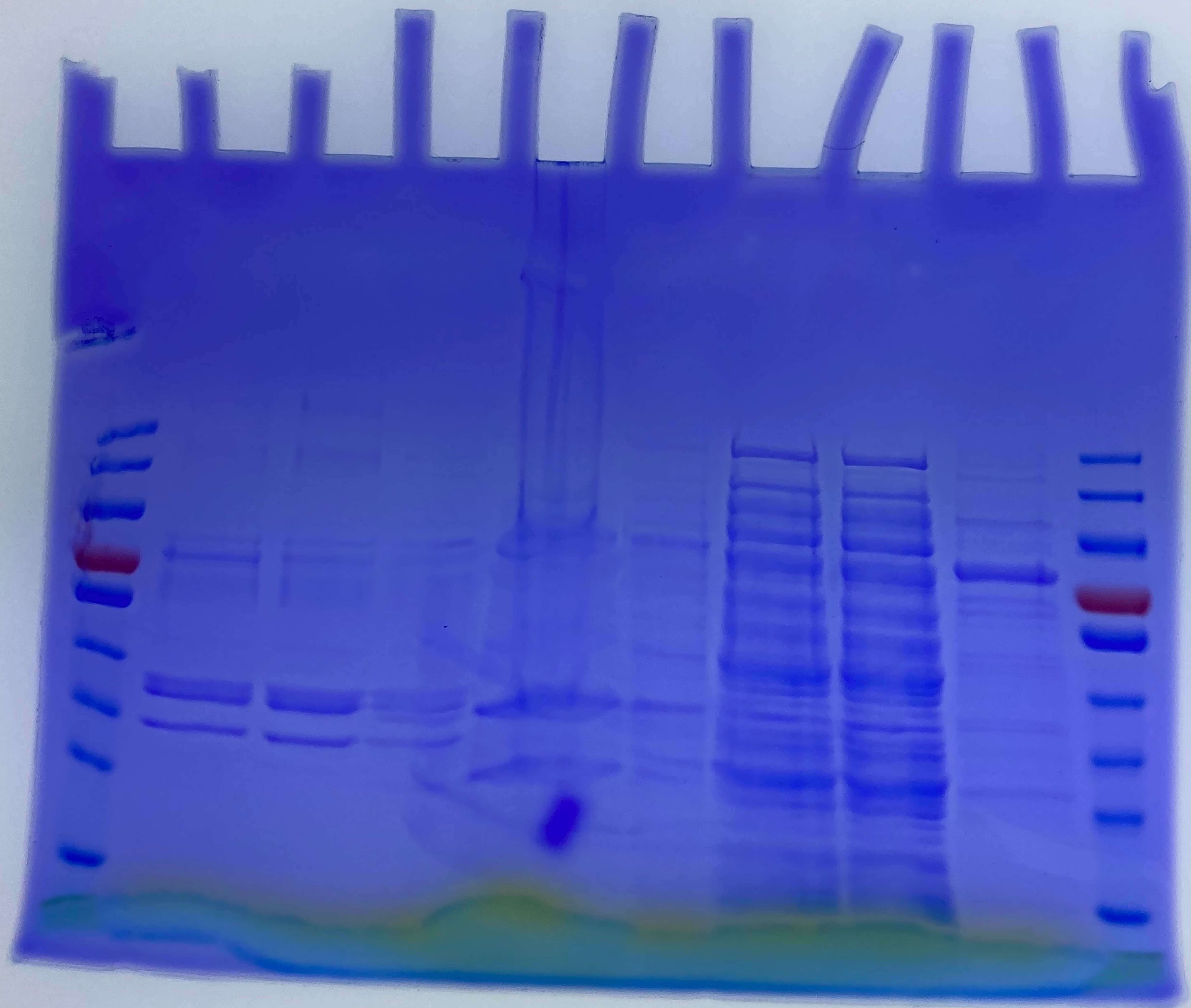

Fig.S2A

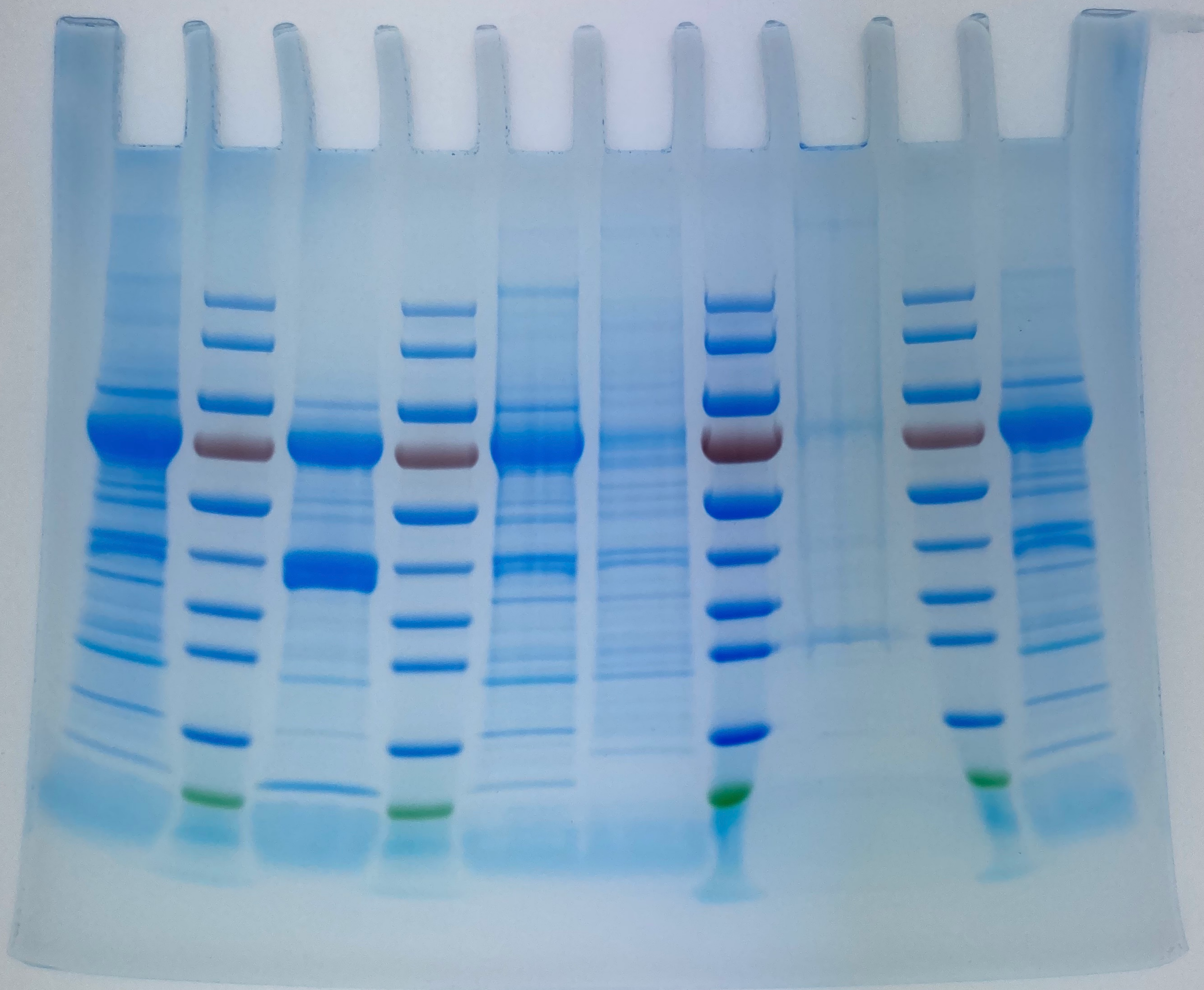

Supplement: S1 Raw Images — (PDF) [file pbio.3002838.s014.pdf]
